# The Proteomic Profiling of Circulating Extracellular Vesicles of Western Diet and Chemical‐Induced Murine MASH Model

**DOI:** 10.1002/kjm2.70107

**Published:** 2025-09-09

**Authors:** Szu‐Jen Wang, Yung‐Ho Wang, Jee‐Fu Huang, En‐Sheng Lin, Wei‐Shiun Chen, Chia‐Yang Li, Chia‐Yen Dai, Wan‐Long Chuang, Ming‐Lung Yu, Shu‐Chi Wang

**Affiliations:** ^1^ Graduate Institute of Clinical Medicine Kaohsiung Medical University Kaohsiung Taiwan; ^2^ Division of Hepatogastroenterology, Department of Internal Medicine Shin Huey Shin Hospital Kaohsiung Taiwan; ^3^ Division of Gastroenterology, Department of Internal Medicine Yuan's General Hospital Kaohsiung Taiwan; ^4^ School of Medicine Kaohsiung Medical University Kaohsiung Taiwan; ^5^ Hepatobiliary Division, Department of Internal Medicine and Hepatitis Center Kaohsiung Medical University Hospital, Kaohsiung Medical University Kaohsiung Taiwan; ^6^ Hepatitis Research Center, College of Medicine; Center for Metabolic Disorders and Obesity; Center for Liquid Biopsy and Cohort Research Kaohsiung Medical University Kaohsiung Taiwan; ^7^ School of Medicine and Doctoral Program of Clinical and Experimental Medicine, College of Medicine and Center of Excellence for Metabolic Associated Fatty Liver Disease National Sun Yat‐sen University Kaohsiung Taiwan; ^8^ Department of Medical Laboratory Science and Biotechnology Kaohsiung Medical University Kaohsiung Taiwan; ^9^ Graduate Institute of Medicine Kaohsiung Medical University Kaohsiung Taiwan; ^10^ Department of Medical Research Kaohsiung Medical University Hospital Kaohsiung Taiwan

**Keywords:** biomarker, extracellular vesicles (EVs), metabolic‐associated steatohepatitis (MASH), metabolic‐associated steatotic liver disease (MASLD), proteomic

## Abstract

Metabolic dysfunction‐associated steatotic liver disease (MASLD) is an increasingly prevalent chronic liver condition that can progress to severe complications such as metabolic dysfunction‐associated steatohepatitis (MASH). Despite its growing burden, there are no reliable non‐invasive biomarkers for tracking disease progression. In this study, we established a murine MASLD/MASH model using a high‐fat diet and chemical (CCl_4_) induction. We analyzed serum‐derived extracellular vesicles (EVs) at 14 and 28 weeks to identify stage‐specific proteomic signatures. Proteomic profiling of circulating EVs revealed key proteins associated with disease progression, including cathepsin B (Ctsb) and prosaposin (Psap) in early MASLD, and coagulation factor XIII A chain (F13a1) and polymeric immunoglobulin receptor (Pigr) in early MASH. The significant and severe MASH stages notably enriched Psma2, Psmb3, and Psmb5. These findings suggest EV‐associated proteins may be promising non‐invasive biomarkers for differentiating MASLD/MASH stages and guiding clinical monitoring.

## 1 Introduction

Metabolic dysfunction‐associated steatotic liver disease (MASLD), formerly known as nonalcoholic fatty liver disease (NAFLD), is characterized by excessive fat accumulation in the liver, often associated with overweight/obesity and metabolic dysfunctions. It has become a major global cause of chronic liver disease that affects approximately 25% of the global population [1, 2, 3]. MAFLD represents a spectrum of liver conditions, ranging from simple steatosis to metabolic dysfunction‐associated steatohepatitis (MASH) [4]. MASH, the severe form of MAFLD, is characterized by hepatic inflammation and injury, including liver ballooning. Without treatment, MASH can progress to cirrhosis and hepatocellular carcinoma (HCC), posing significant health risks and is anticipated to become a leading cause of liver transplantation [5].

Extracellular vesicles (EVs), including small extracellular vesicles (sEVs) like exosomes and microvesicles, are membrane‐bound nanoparticles released by various cell types under both normal and pathological conditions [6], such as liver disease [7]. Small EVs (sEVs), measuring approximately 30–150 nm in diameter, carry bioactive substances, such as proteins and nucleic acids, that can modulate recipient cell metabolism [8]. Lipotoxic hepatocyte‐derived sEVs enriched with proteins and miRNAs are absorbed by hepatic stellate cells (HSCs), promoting hepatic fibrosis progression [9, 10]. Adipocyte‐derived EVs (adipocyte‐EVs) also play critical roles in NAFLD pathogenesis, distinct from traditional adipokines like adiponectin and leptin, by delivering specific bioactive cargos that regulate recipient cell function [11]. Additionally, damaged hepatocytes release hepatocyte‐EVs (Hep‐EVs), which activate liver sinusoidal endothelial cells, HSCs, and hepatic macrophages, exacerbating liver damage in diseases like MASH [12, 13]. EVs enable intercellular communication by transferring molecular cargo that reflects the donor cell's state to target cells, influencing healthy and pathological processes, including liver fibrosis and disease progression [14, 15]. Increasing evidence highlights liver‐derived EVs as key mediators in liver disease pathogenesis, making them potential targets for future research and therapeutic interventions [16, 17]. Exploring the key fibrotic factors that shuttle from sEV to HSCs is crucial for understanding MAFLD‐related disease progression.

Liver biopsy remains a fundamental tool for diagnosing and managing liver diseases, including MAFLD and MASH [18]. Despite its diagnostic significance, this procedure is invasive, expensive, and prone to sampling errors and complications, burdening patients considerably [19, 20]. While non‐invasive biomarkers are available, their limited accuracy often necessitates combination with other markers for reliable diagnosis [21]. These limitations highlight the urgent need for alternative and more effective diagnostic methods. Liquid biopsy has emerged as a cutting‐edge diagnostic approach, offering superior accuracy, speed, repeatability, affordability, and non‐invasive advantages over traditional liver biopsy methods [22]. This technique facilitates the isolation of EVs directly from blood samples. EVs, membrane‐bound particles released by cells, contain diverse bioactive cargos such as proteins, mRNA, metabolites, and lipids, making them promising biomarkers for disease progression [23]. This study focuses on isolating serum‐derived EVs and conducting proteomic analysis to discover novel non‐invasive biomarkers for diagnosing and monitoring different stages of MASLD/MASH.

## 2 Materials and Methods

### 2.1 Establishment of Experimental Animal Models to Simulate MASLD/MASH Progression

The Kaohsiung Medical University Animal Center ensured a controlled environment for the male C57BL/6 mice, maintaining specific conditions including a temperature of 21°C ± 2°C, relative humidity of 55%–70%, and a 12‐h light/dark cycle. Ethical standards were rigorously followed, with approval from the Institutional Animal Care and Use Committee of Kaohsiung Medical University, Taiwan, per established guidelines for laboratory animal care. To establish the MASLD/MASH animal model, a modified protocol based on previous studies was utilized [24]. Mice, aged 7 weeks and weighing 23 ± 2 g, were procured from Taiwan's National Laboratory Animal Center and acclimated for 1 week before the experiment. They were randomly assigned to two main feeding groups, with durations of 14 and 28 weeks. Each feeding group was further subdivided into four dietary treatment subgroups: normal diet (ND), western diet (WD), ND with Tetrachloromethane (CCL_4_) injection (ND + CCL_4_), and WD with CCL_4_ injection (WD + CCL_4_). ND mice were fed Altromin 1310 and double‐distilled water (ddH_2_O), while WD mice received Envigo Teklad custom diet (TD.120528) with syrup containing 2.31% fructose and 1.89% glucose to induce MASLD/MASH. The mice were administered CCL_4_ (2%) at a dosage of 1/100 of their body weight. Weekly weighing was conducted for all groups, and mice were sacrificed at 14 and 28 weeks to collect organ and blood samples for further analysis.

### 2.2 Biochemical Analysis

Blood samples obtained from the mice were centrifuged at 500 g for 15 min at 4°C. Serum glucose levels, intraperitoneal glucose tolerance (IGTT), total cholesterol (TC), and triglycerides (TG) were quantified using the FUJIFILM DRI‐CHEM NX‐500 system.

### 2.3 Histopathological Staining and Fibrosis Quantification

Liver tissues, fixed and sectioned, were subjected to hematoxylin and eosin staining, Picro‐Sirius Red staining, and Masson staining. A pathologist conducted a blinded evaluation of the specimens using a light microscope, adhering to the NASH CRN Scoring System [25]. Steatosis and fibrosis were quantified using Image J software, which measured cell vacuolation and proportions of red fibrotic tissue [26].

### 2.4 Extracellular Vesicles (EVs) Isolation

EVs were isolated from the serum specimens collected from sacrificed C57BL/6 mice using the ExoQuick‐TC protocol [27]. Initially, serum samples were centrifuged at 3000 × *g* for 15 min at 4°C to remove cells and debris. The resulting supernatant was transferred into sterile tubes and mixed with ExoQuick‐TC at 1:5 (serum:ExoQuick‐TC). The mixture was refrigerated overnight at 4°C. Following incubation, the samples were centrifuged at 1500 × g for 30 min at 4°C, resulting in EV pellets at the bottom of the tubes. After aspirating the supernatant, the pellets underwent another centrifugation at 1500 × *g* for 5 min at 4°C. Finally, the residual fluids were removed, and the pellets were resuspended in 1X sterile PBS buffer within Eppendorf tubes, yielding purified EVs.

### 2.5 Sample Preparation

The proteins were extracted and subjected to analysis using 10% SDS‐PAGE (1 cm). After excising the gel spot, de‐staining was performed, followed by reduction with 10 mM dithiothreitol (DTT, Merck) at 60°C for 45 min. Cysteine residues were blocked using 55 mM iodoacetamide (IAM, Sigma) at 25°C for 30 min. The samples were digested overnight at 37°C with sequencing‐grade modified porcine trypsin (Promega). Subsequently, peptides were extracted from the gel, dried via vacuum centrifugation, and reconstituted in 0.5% formic acid for further analysis.

### 2.6 Liquid Chromatography‐Mass Spectrometry (LC‐MS/MS) Analysis

Digested peptides were diluted in HPLC buffer A (0.1% formic acid) and loaded onto a reverse‐phase column for separation using a multi‐step gradient of HPLC buffer B (99.9% acetonitrile/0.1% formic acid) at a flow rate of 0.3 μL/min over 70 min. The separation was performed on an Orbitrap Elite ETD mass spectrometer, with full‐scan MS conducted at a resolution of 120,000 over 400 to 2000 Da. Internal calibration used the ion signal at m/z 536.165365 as a lock mass. Twenty MS/MS scans were acquired for the most abundant precursor ions, dynamically excluded for 40 s with a mass tolerance of 15 ppm.

### 2.7 Protein Identification

Proteome Discoverer software (version 1.4, Thermo Fisher Scientific) was used for data analysis. MS/MS spectra were searched against the SwissProt database using the Mascot search engine (version 2.5, Matrix Science, London, UK). Peptide identification was conducted with a 10 ppm mass tolerance for intact peptide masses and 0.5 Da for CID fragment ions, allowing for two missed cleavages during trypsin digestion. Variable modifications included oxidized methionine and acetylation (protein N‐terminal), while carbamidomethylation (cysteine) was a static modification. Peptide‐spectrum matches (PSMs) were filtered based on high‐confidence criteria and rank one identification by the Mascot search engine, ensuring an overall false discovery rate below 0.01. Proteins identified with a single peptide hit were retained for further analysis.

### 2.8 Proteomic Analysis of EVs

Mass spectrometric analysis was performed using a matrix‐assisted laser desorption/ionization time‐of‐flight mass spectrometry (MALDI‐TOF‐MS) system (Autoflex III Smartbeam, Bruker Daltonics, Billerica, MA, USA) equipped with a 355 nm Nd: YAG laser [Huang, 2015 #33]. To investigate EV‐associated proteins relevant to different stages of MASLD and steatohepatitis (MASH), peptide sequence data were acquired and analyzed. Proteins were initially screened based on peptide spectrum matches (PSMs), and those with increased expression were selected from the WD and WD with carbon tetrachloride (WD + CCl_4_) groups at both 14 and 28 weeks. Protein sequences from all four experimental groups at each time point were compared to identify proteins specifically associated with disease progression. Shared proteins were excluded to isolate those uniquely expressed in the WD and WD + CCl_4_ groups. The curated protein lists were submitted to the STRING database for functional interaction and enrichment analysis [28].

## 3 Results

### 3.1 Establishment of Animal Models to Simulate MASLD Progression to Significant MASH Through a 14‐ and 28‐Week Feeding Regimens

**FIGURE 1:**
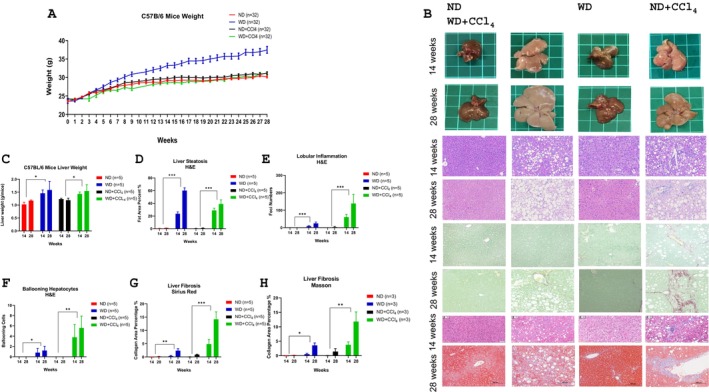
Development of the MASLD/MASH progression animal model using a normal or western diet, with or without CCl4 administration. (A) body weight changes, (B) liver morphology and histopathology with H&E, Sirius Red, and Masson stains, (C) liver weight, (D) steatosis, (E) lobular inflammation, (F) ballooning, and (G, H) fibrosis levels. Comparisons are drawn between groups of mice fed a normal diet (ND), a western diet (WD), ND with CCl4, and WD with CCl4 for durations of 14 and 28 weeks (*n* = 5 per group). The presented data represent means ± SD from three independent experiments. Statistical analysis utilized ordinary one‐way ANOVA with Bonferroni's multiple comparison tests. *p < 0.05; **p < 0.01; ***p < 0.001. MASH—metabolic dysfunction‐associated steatohepatitis; MASLD—metabolic dysfunction‐associated steatotic liver disease; ND—normal diet; WD—western diet.

After 6 weeks of WD feeding, the WD group exhibited significant weight gain compared to the ND, ND + CCL_4_, and WD + CCL_4_ groups (Figure 1A). Additionally, liver weight was consistently higher in the WD and WD + CCL_4_ groups throughout the 14‐ and 28‐week feeding periods compared to the ND and ND + CCL_4_ groups (Figure 1B,C). Liver histopathology of 14‐week WD mice showed pronounced steatosis, with no lobular inflammation, ballooning, or fibrosis, representing early MASLD. In contrast, histopathology of 28‐week WD mice demonstrated early MASH, characterized by significant steatosis, mild lobular inflammation, ballooning, and fibrosis. The histopathological analysis of WD + CCL_4_ mice demonstrated severe steatosis, lobular inflammation, hepatocyte ballooning, and fibrosis, accompanied by substantial fibrous tissue accumulation forming septa in 28‐week WD + CCL_4_ mice. These findings indicate MASH development in 14‐week WD + CCL_4_ mice and its progression to advanced MASH, approaching cirrhosis, by 28 weeks (Figure 1B–1H). These findings accurately represent the progression from early MASLD in 14‐week WD mice to early MASH in 28‐week WD mice; the WD + CCL_4_ group represents the development of significant MASH in 14‐week WD + CCL_4_ mice and its progression to cirrhosis in 28‐week mice.

### 3.2 Exploring Potential Biomarkers for MAFLD/MASH Through EV Proteomics

We performed proteomic analyses on EVs derived from our animal model to discover novel non‐invasive biomarkers for the various stages of MASLD/MASH. After evaluating the specimens, protein analyses were conducted at two time points: the mid‐point (14 weeks) and the late‐point (28 weeks) across four experimental groups. EVs were isolated using ExoQuick‐TC and analyzed via LC‐MS/MS to determine their proteomic profiles.

**FIGURE 2:**
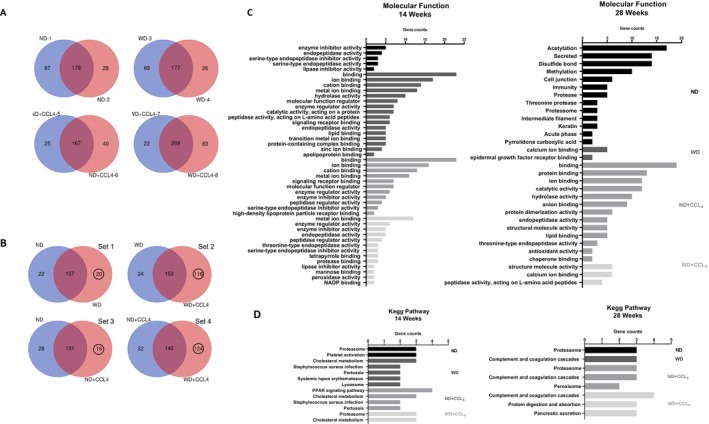
Comparative proteomic profiling reveals distinct and overlapping protein expression patterns across dietary and fibrotic mouse models. (A) Intragroup consistency analysis by Venn diagrams represents the overlap of proteins identified in biological replicates within each group: ND (normal diet), WD (western diet), ND + CCL_4_ (normal diet with carbon tetrachloride‐induced liver injury), and WD + CCL_4_. (B) Intergroup comparisons by Venn diagrams illustrate the number of shared and unique proteins between the four experimental groups. (C) Molecular function analysis by Gene Ontology (GO) enrichment. Bar graphs show significantly enriched GO terms associated with molecular functions for differentially expressed proteins at 14 and 28 weeks. (D) KEGG pathway enrichment analysis. Differentially expressed proteins were mapped to KEGG pathways. At 14 and 28 weeks, several pathways were significantly enriched: metabolic reprogramming and fibrosis‐associated pathway activation.

To ensure the validity and reliability of the protein and gene data, protein lists were uploaded to the Draw Venn Diagram tool (http://bioinformatics.psb.ugent.be/webtools/Venn/) to calculate intersections. As illustrated in Figure 2A, the intersections within the same group constituted a significant proportion (approximately 65%–72%), confirming the credibility of proteins extracted from each group of mice. Next, to identify the group most relevant to MASLD, proteins from each group were intersected using the Venn Diagram tool. This yielded four distinct protein data sets (Figure 2B): no CCL_4_ under WD diet (set 1), CCL_4_under WD diet (set 2), ND diet under CCL_4_ (set 3), and WD diet under CCL_4_ (set 4). Notably, the CCL4 under the WD diet and the WD diet under the CCL4 groups contained 116 and 124 unique proteins, respectively, which may play crucial roles in MASH development. Additionally, protein sequences identified via Proteome Discoverer software were further analyzed for molecular functions and KEGG pathways using STRING [28] (Figure 2C,D).

**FIGURE 3:**
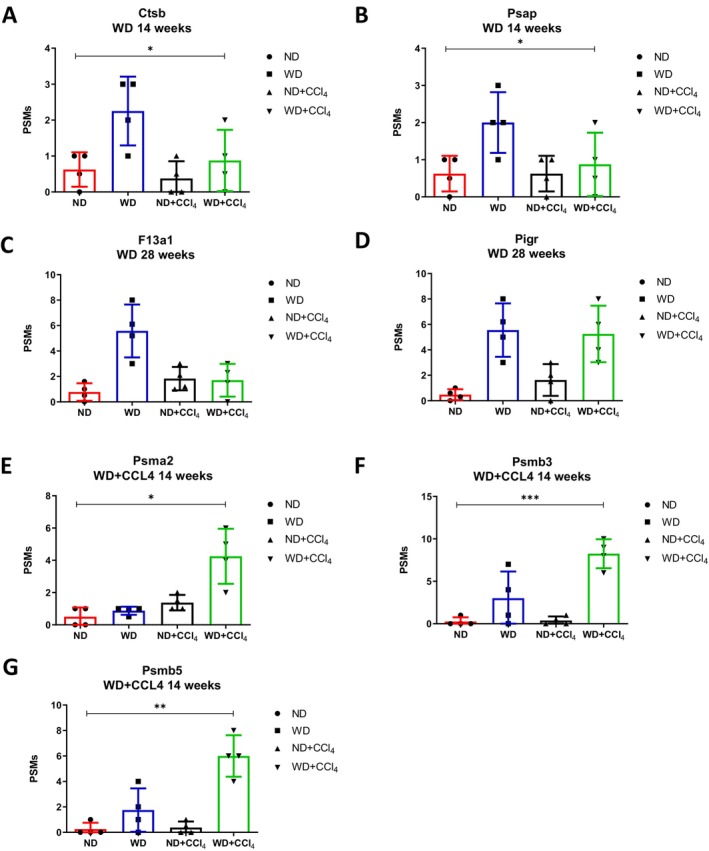
Representative expression profiles of common proteins elevated in the WD and WD + CCL4 groups at 14 and 28 weeks. (A, B) Expression of Ctsb and Psap protein levels in liver tissues from the WD group at 14 weeks. (C–E) Expression of Psma2, Psmb3, and Psmb5 in the WD + CCL4 group at 14 weeks. (F, G) Expression levels of F13a1 and Pigr proteins in the WD group at 28 weeks. Statistical significance is indicated as follows: ***p < 0.001, **p < 0.01, *p < 0.05.

The goal was to establish a link between the EV‐derived proteome and different MASLD/MASH stages. Based on weight change data and histopathological analyses (Figure 1), we categorized the stages as follows: the WD group at 14 weeks represented the early MASLD, while the WD group at 28 weeks reflected the early MASH. Meanwhile, the WD + CCL_4_ group at 14 weeks indicated significant MASLD, and at 28 weeks, this group progressed to severe MASH. Proteomic analysis revealed potential biomarkers, including increased and unique proteins within EVs from the WD/WD + CCL_4_ groups at both time points. Common proteins across the groups were initially identified based on peptide‐spectrum match (PSM) scores. For the WD group, higher PSM scores were found in proteins Ctsb (Figure 3A) and Psap (Figure 3B) at 14 weeks, as well as F13a1 (Figure 3C) and Pigr (Figure 3D) at 28 weeks. In the WD + CCL_4_ group, elevated PSM scores were observed in Psma2 (Figure 3E), Psmb3 (Figure 3F), and Psmb5 (Figure 3G) at 14 weeks, while no common proteins showed higher PSM scores at 28 weeks.

Next, unique proteins were identified by intersecting all protein sequences across groups to exclude common proteins. These unique proteins from the WD/WD + CCL_4_ groups at both 14 and 28 weeks are listed in Table 1. With these findings, we established connections between EV‐derived proteomics and distinct MASLD/MASH stages, unveiling promising new biomarkers for early MASLD (WD group, 14 weeks), early MASH (WD group, 28 weeks), significant MASLD (WD + CCL_4_ group, 14 weeks), and severe MASH (WD + CCL_4_ group, 28 weeks) (Figure 3, Table 1).

**TABLE 1:** Unique proteins identified in WD mice and WD with CCl4 mice for durations of 14–28 weeks. Proteins exclusively detected in western diet (WD) and WD with carbon tetrachloride (CCl_4_) treatment groups, categorized by 14 weeks and 28 weeks. Protein identifiers and gene symbols are provided for the list.

Unique genes from each group, 14 weeks	Group	Gene symbol	Unique genes from each group, 28 weeks	Group	Gene symbol
	WD	Piga Zfp90 Nrp1 Filip1 Lypla1 Mmrn1 Myo1h Trf Angptl3 Igj Krt14 Fbp1 Aldob Dclre1b Krt1 Pcyox1 Krt76 Chd1 Pmpca Saa1 Pik3r2 Grsf1 Prss1 Ndufb7 Mup20 Obp1a		WD	Hgd Gnmt Bhmt Comp Lifr Ccdc25 Syngap1 Ppip5k1 Me1 Ywhaz Il1rap Aldob Psmb8 Abcd2 Ldhc Mup2 H2‐Q8 Rhof Fer Enpp2 Phtf1 Arpc3 Serpina7 Ca3
	WD + CCL4	Ttc41 Polr3a Ltf Psma3 Bsg Actr5 Psmb8 Cwc25 Creb3 Clspn Pomgnt1		WD + CCL4	Proz Slc1a1 Amy1 Trmt11 Myo9a Dclre1b Trio Slc15a4 Krt73 Krt76 Trpc4 Iglc3 Ccdc30 Hemk1

## 4 Discussion

Given the rising prevalence of MASLD and the lack of effective monitoring methods for MASLD/MASH progression, there is an urgent need for new non‐invasive biomarkers. Compared to traditional liver tissue biopsy—an invasive procedure with several limitations [29]—non‐invasive biomarkers offer a faster, safer, serial, and cost‐effective approach for detecting different stages of MASLD/MASH. Circulating EVs, which carry various biomolecules, provide a promising non‐invasive means of monitoring liver disease [30]. This study explored the relationship between proteins in EVs and the different stages of MASLD/MASH to identify novel non‐invasive biomarkers. Using our animal model, four groups of C57BL/6J mice were subjected to distinct dietary regimens and treatments over 14 and 28 weeks to induce varying levels of MASLD/MASH. By isolating EVs from the serum of these mice at both time points, we identified increased and unique proteins in the WD and WD + CCL_4_ groups at 14 and 28 weeks.

Carbon tetrachloride (CCl_4_) was selected as a hepatotoxic agent in this study to accelerate liver injury and fibrosis development, mimicking advanced stages of MASH more efficiently. CCl_4_ is a well‐established chemical inducer of liver fibrosis due to its ability to generate reactive oxygen species (ROS) and promote hepatocyte necrosis, inflammation, and stellate cell activation. When combined with a WD, which promotes steatosis and metabolic dysfunction, CCl_4_ enhances the pathophysiological relevance of the model by simulating the transition from early MASLD to advanced MASH and even cirrhosis. This research offers several advantages compared to previous studies on MASLD and EVs. Earlier studies typically established animal models using a single time point and relied solely on dietary interventions [31]. In contrast, our study examined two distinct time points (14 and 28 weeks) and incorporated dietary variations and carcinogen treatments by CCL_4_. By investigating more than one time point, we could observe the progression of MASLD/MASH over time. Exposing the animals to different diets and CCl_4_ allowed us to evaluate MASLD/MASH outcomes induced by high‐fat diets alone and those accelerated by the interaction between high‐fat diets and CCl_4_. This approach provided a more comprehensive understanding of disease progression. Furthermore, the inclusion of CCL_4_ treatment made our experimental design more representative of real‐world conditions, as patients are often exposed to environmental toxins and carcinogens in daily life.

These findings offer substantial translational potential for developing non‐invasive biomarkers to monitor MASLD/MASH progression. Several of the EV‐associated proteins identified in this study, such as cathepsin B (Ctsb), prosaposin (Psap), coagulation factor XIII A chain (F13a1), and the polymeric immunoglobulin receptor (Pigr), are known to be detectable in human serum and have been implicated in metabolic or inflammatory liver conditions. cathepsin B is elevated in the serum of patients with steatohepatitis and hepatocellular carcinoma and plays a role in fibrogenesis and HSC activation ([Liu, 2023 #16]; [Eguchi, 2019 #14]). Prosaposin, a lysosomal protein involved in lipid metabolism, has been reported as a circulating biomarker in metabolic diseases ([Zhang, 2024 #32]). F13a1 is a coagulation‐related protein measurable in plasma and has been associated with liver fibrogenesis and extracellular matrix stabilization ([Newman, 2022 #30]). Similarly, Pigr is a marker of epithelial immune response and has been found elevated in liver inflammation ([Povero, 2015 #12]). Moreover, proteasome subunits such as Psma2, Psmb3, and Psmb5, while traditionally studied in oncology and systemic inflammation, are emerging as plasma biomarkers of tissue damage and proteostasis, including in liver disease ([Dorairaj, 2020 #8]). These proteins likely originate from both hepatic and extrahepatic sources, reflecting the complex pathophysiology of the disease. Notably, cathepsin B (Ctsb) and prosaposin (Psap) were elevated in EVs from early MASLD (WD 14‐week), suggesting hepatocyte or macrophage activation under lipotoxic stress. In contrast, F13a1—a coagulation factor associated with extracellular matrix stabilization—was enriched during early MASH (WD 28‐week), implicating HSC involvement in fibrogenesis. The unique presence of proteasomal subunits (Psma2, Psmb3, Psmb5) in the WD + CCL4 group at 14 weeks further supports their potential as biomarkers of significant hepatocellular stress and early fibrotic remodeling. While these proteins are predominantly liver‐derived, detecting proteins like Pigr also suggests contributions from mucosal or immune‐related tissues, highlighting the systemic nature of MASLD/MASH. Thus, our findings underscore the diagnostic value of EV proteomics in capturing the dynamic interplay between hepatic and extrahepatic responses during disease progression.

EV‐associated proteins hold strong potential as non‐invasive biomarkers for the development and progression of MASLD/MASH [32]. However, the identified protein signatures remain limited in scope and are derived from a single murine model. Further validation is essential to establish their specificity and reproducibility. Not all detected proteins may be uniquely linked to MASLD/MASH pathogenesis, while effective in accelerating fibrosis by CCl_4_, may introduce non‐metabolic injury mechanisms that differ from the natural course of MASLD/MASH in humans, thereby limiting direct translational relevance; some could reflect non‐specific systemic or strain‐dependent effects, particularly in the C57BL/6J mouse model. To strengthen the translational potential of our findings, future studies will focus on EV subtype‐specific profiling—distinguishing exosomes, microvesicles, and other vesicle types—to increase the precision and biological relevance of candidate biomarkers. Fractionating and characterizing EV subpopulations using established surface markers will help refine disease‐specific proteomic signatures and minimize background noise. Ultimately, the study aims to provide clinically applicable, EV‐based biomarker panels capable of monitoring MASLD/MASH progression to severe fibrosis, therapeutic response, and risk stratification. Achieving this will require integrative validation in multiple disease models, human cohorts, and advanced proteomic and bioinformatic approaches to ensure biomarker robustness and clinical utility.

## 5 Conclusion

This study highlights EV‐associated proteins as potential non‐invasive biomarkers for MASLD/MASH progression. Using an animal model with dietary and carcinogen treatments, we identified disease‐stage‐specific proteins, offering an alternative to liver biopsies. Further validation is required for clinical application.

## Conflicts of Interest

Ming‐Lung Yu received a research support (grant) from BMS, Gilead, Merck, and Roche diagnostics; is a consultant of AbbVie, BMS, Gilead, Roche, and Roche diagnostics; and is a speaker of AbbVie, BMS, Eisai, Gilead, Roche and Roche diagnostics. Wan‐Long Chuang is an advisory board member for AbbVie, BMS, Gilead, PharmaEssentia, Roche, and Vaccitech; is a speaker for AbbVie, BMS, Gilead, and Roche. Chia‐Yen Dai/Chung‐Feng Huang is a speaker for AbbVie, BMS, Gilead, Merck, and Roche. The other authors declare no conflicts of interest.

## Data Availability

The data that support the findings of this study are available from the corresponding author upon reasonable request.
